# Molecular Signatures of Radiation Response in Breast Cancer: Towards Personalized Decision-Making in Radiation Treatment

**DOI:** 10.1155/2017/4279724

**Published:** 2017-11-26

**Authors:** Corey Speers, Lori J. Pierce

**Affiliations:** ^1^Department of Radiation Oncology, Michigan Medicine, Ann Arbor, MI, USA; ^2^Comprehensive Cancer Center, University of Michigan, Ann Arbor, MI, USA

## Abstract

Recent advances in gene expression profiling have allowed for a more sophisticated understanding of the biology of breast cancers. These advances led to the development of molecular signatures that now allow clinicians to more individually tailor recommendations regarding the utility and necessity of systemic therapies for women with breast cancer. Indeed, these molecularly based tests have been incorporated into national and international best practice guidelines and are now part of routine practice. Similar, though slower, progress is being made in the development of molecular signatures predictive of radiation response and necessity for women with breast cancer. This article will discuss the history of radiation response signature development, the current state of these signatures under ongoing clinical development, the barriers to their clinical adoption, and upcoming changes and opportunities that may allow for the personalized radiation treatment recommendations enabled by the development of these signatures.

## 1. The History of Radiation Response Signature Development

The idea that genes (either DNA sequence or subsequent expression) may function as surrogate biomarkers of disease response forms the rationale for the development of molecularly based signatures to predict response to radiation treatment in breast cancer. Prior to the genomics era and for over half a century, the field relied upon the response of* in vitro* cultured normal and malignant cells to radiation as the basis for models describing the radiation response of* in vivo* malignancies [1–7]. While beyond the scope of this review, these experiments provided the foundational data upon which the linear-quadratic model was derived, but the data derived from these experiments was incomplete in its ability to adequately model the heterogeneous response of tumors and normal tissue to ionizing radiation. Given these limitations, investigators have broadened their interrogation to try to develop more effective ways of both describing and predicting the response of tumors to radiation treatment. This search, coupled with technological advances that allow for the more complete evaluation of DNA, RNA, protein, and cellular metabolism, has led to the development of “-omic” based approaches for the prediction of response of tumors to treatment [8–15].

Pioneering work in the area of genomic-based signature development focused on prognostication and response to systemic therapy [16–22]. These early investigations focused on using genomic-based approaches to predict response to chemotherapy and/or hormone therapy, or to determine prognosis if adjuvant therapies were altogether omitted after surgery. Indeed, several genomic assays are currently in clinical practice that function either as prognostic biomarkers to aid in determining prognosis independent of treatment or as predictive biomarkers that are useful in directing appropriate clinical management. Initially these genomic tests were restricted to the expression or mutation of a single gene. More recently, as sequencing and high-throughput assaying techniques have improved, these genomic tests have become more sophisticated and their indications for utilization have increased. With respect to breast cancer prognostic and predictive biomarkers, OncotypeDx®, Prosigna™, MammaPrint®, EndoPredict, Mammostrat®, and Breast Cancer Index all represent genomic tests with either prognostic or predictive capability that aid in risk stratification beyond standard clinicopathologic parameters in breast cancer [20, 21, 23–28]. While each test varies in its clinical indication, utility, and genomic make-up, many have been successfully validated as having clinical utility and have been adopted, to varying degrees, into clinical practice. Indeed, some of these tests are now part of recommended work-up by the national and international “best practice” guidelines as set forth by the National Comprehensive Cancer Network (NCCN) and other professional societies.

The concept of using genomic-based approaches for prediction of systemic therapy response is not unique. Although similar genomic tests for the prediction of tumor response after radiation have been slower in development, within the last several years an increasing number of tests with varying clinical indications and utility have been reported [9, 11, 14, 15, 29, 30]. Numerous groups have described gene expression-based genomic tests that predict the likelihood of breast cancer response to radiation treatment (or lack thereof) in an effort to identify the patients either (A) likely to respond (or not respond) to adjuvant radiation treatment or (B) not needing adjuvant radiation treatment at all. Most of these groups utilized high-throughput RNA expression technologies to develop gene expression signatures prognostic of low local recurrence risk (for the prognostic signatures) or predictive of response to radiation treatment in the adjuvant setting for patients with early-stage disease.

## 2. Radiation-Induced Gene Signatures

The concept of utilizing gene expression changes induced by radiation exposure as the basis for molecular signature development was foundational to the development of a number of signatures, either predictive or prognostic, for outcomes of patients with breast cancer. These signatures vary widely in the methods employed for their development, as well as their specificity for breast cancer outcomes and relationship to radiation treatment. Given this heterogeneity in development and validation, it is not surprising, then, that there is not significant overlap in the genes associated with these signatures. Additionally, the external validation of these signatures has been challenging for a number of reasons that will be discussed later in this article.

In 2009, Piening et al. described the derivation of signature that was developed based on the change of gene expression in human lymphoblast cells from 12 persons' cells exposed to 5 Gy of Cesium radiation [31]. When compared to unirradiated cell controls, the subsequent significantly induced (160 genes) and repressed (59 genes) genes were used as the basis of a prognostic signature that was applied to 2 independent breast cancer cohorts. While the datasets were not derived from breast cancer patients, the authors found that the application of, and supervised hierarchical clustering in, these two breast cancer datasets did indeed have prognostic import. Unsupervised hierarchical clustering broadly identified two clusters of patients and when Kaplan-Meier survival analysis was employed, the radiation-induced and radiation-repressed gene signatures were significantly associated with local recurrence. In addition, the authors identified biological pathways associated with radiation response, including p53 responsive and proliferative genes.

Another early predictive signature was an interferon-based signature that was developed to predict response not only to ionizing radiation, but also to DNA-damaging chemotherapies [13]. This group developed the interferon-related DNA damage resistance signature based on genes associated with radiation resistance in a single squamous cell carcinoma cell line model and subsequently demonstrated that this signature was predictive of response to chemotherapy and ionizing radiation in women with breast cancer. They further refined the signature to include a 7-gene-pair classifier (from the original panel of 49 differentially expressed genes) that predicted the efficacy of adjuvant chemotherapy as well as local control after radiation treatment. Despite this early development, subsequent translation into a clinically relevant test has not been detailed.

Another group simply applied previously developed molecular signatures which were either prognostic of outcomes for metastasis-free or overall survival endpoints or predictive of response to systemic chemotherapy in breast cancer and evaluated the utility of these signatures to predict locoregional disease control. Although many of these previously developed signatures proved to be able to predict local control after radiation treatment, a previously developed “wound response” signature was found to be most prognostic of locoregional disease control in women treated with postoperative radiation therapy [32].

In addition to radiation-induced gene response signatures, other groups have looked at signatures that may predict response to radiation treatment, though not necessarily for breast cancer. These signatures include hypoxia-related signatures [33–35], cell cycle and DNA damage gene related signatures [36, 37], and signatures predictive of response to radiosensitizing drugs [38–40]. As with many of the other* in vitro* derived signatures, none of these signatures has thus far withstood the rigors of external validation and thus has not yet been translated into the clinic.

## 3. Radiation Intensification Signatures

While intriguing at the basic biology level, as noted previously these radiation-gene induced signatures have not been readily translated into clinically useful tests for women with breast cancer. Other groups have taken an alternative approach to identify patients who need treatment intensification. Indeed, a potential additional use of molecularly based signatures is to identify women for whom standard trimodality therapy (surgery, chemotherapy, and/or radiation) is insufficient. If these signatures were significantly robust in identifying women likely to fail despite radiation treatment (because of, e.g., radioresistance inherent in the tumor), more aggressive or alternative treatment strategies might be employed. One of the first potentially useful signatures was developed by investigators in Sweden. They analyzed gene expression differences between 143 early-stage breast cancer patients all of whom were treated with radiation after surgery to identify gene signatures prognostic of local recurrence in patients treated with radiation [41]. This group identified an 81-gene signature (the 81 genes being selected as the “most strongly associated” with recurrence) and found that this signature could outperform clinical and pathologic characteristics alone for prognostication of local recurrence events. Another similarly designed study from Dutch investigators performed gene expression microarray profiling to determine differently expressed genes between tumors from women who developed local recurrence after breast conserving surgery and radiation and women who did not [25]. In this institutional study, 56 primary invasive breast carcinomas from patients with a local recurrence were profiled and compared to 109 tumors from patients who did not experience a local recurrence. This study identified over 7,000 significantly regulated genes and performed supervised and unsupervised hierarchical clustering to further refine the list to 104 genes associated with recurrence [25]. This list was significantly enriched for genes involved in cell proliferation, and then a subsequent classifier of local recurrence was built using a 111-gene list that was shown to be independently associated with local recurrence using a multivariate Cox regression analysis. Despite early potential and enthusiasm, both the Swedish and the Dutch signatures failed external validation in datasets in which they were not trained, and other investigators have reported a similar inability to derive a radiation response signature using gene expression from patient tumors as the basis of their signature development [10, 42]. Other investigators have focused on the development of prognostic signatures in breast cancer that are associated with local recurrence after surgery alone and which are independent of radiation treatment, but, like the Swedish and Dutch counterparts, these signatures have not performed well in external validation [10]. A different approach was employed by investigators at the University of Michigan who used breast cancer cell line expression and radiation response data as the basis for the development of a radiation response signature [11]. This radiation response signature was found to be independent of breast cancer intrinsic subtype, and when applied to two independent breast cancer cohorts of patients with breast cancer treated with breast conserving surgery and radiation treatment, the signature was able to identify patients likely to recur despite surgery and radiation and may also have the potential to identify women for whom radiation is altogether unnecessary. The resulting signature, termed RadiotypeDx®, to connote potentially similar application for radiation as OncotypeDx is currently used for systemic therapy, has been externally validated in additional datasets, and now is being studied in previously completed phase III randomized trials in low-risk, early-stage breast cancer patients treated with breast conserving surgery randomized to +/− radiation treatment. While encouraging, this signature also will require true external validation either as part of a prospective randomized trial or with “prospective retrospective” analyses from previous phase III trials before it can be adopted into clinical practice.

## 4. Radiation Omission Signatures

While many signatures have been developed aimed at predicting the sensitivity to, or efficacy of, radiation in breast cancer, data from the Early Breast Cancer Trialists' Collaborative Group (EBCTCG) meta-analysis suggests that the majority of women with early-stage breast cancer treated with breast conserving surgery are cured of their disease with surgery and endocrine therapy alone [43]. Thus, the search for a signature that may identify these extremely low-risk women for whom radiation treatment is unnecessary has been an area of active interest. While there are no true “radiation omission” molecular signatures currently reported for women with invasive breast cancer, numerous groups are working to validate previously derived molecular classifiers as sufficient surrogates for appropriate radiation treatment omission. Indeed, there are four ongoing observational or investigational trials looking at OncotypeDx (IDEA study), Prosigna (PRECISION trial), IHC (LUMINA trial), and IHC4 (PRIMETIME trial) scores as sufficient stratification methods for radiation omission. The Individualized Decisions for Endocrine Therapy Alone (IDEA) study is an investigational trial testing whether an OncotypeDX recurrence score ≤ 18 is sufficient to identify women who may safely omit radiation treatment. In this study with a target accrual of 200 patients, postmenopausal women between the ages of 50 and 69 with early-stage disease will enroll on a trial that omits radiation treatment for women with the low (≤18) OncotypeDx score. These women will receive adjuvant endocrine therapy, and rates of locoregional recurrence, metastasis, and overall survival as well as type of salvage therapy will be tracked, with follow-up planned for 10 years and the primary endpoint being 5-year locoregional recurrence-free survival. In a similarly designed phase II trial, the Profiling Early Breast Cancer for Radiotherapy Omission (PRECISION) trial is looking at whether women 50–75 years of age with a low-risk score based on Prosigna testing are safely able to omit adjuvant radiation after breast conserving surgery. This trial will study the outcomes of 1380 patients with low-risk scores treated with lumpectomy and endocrine therapy without radiation; 5-year rates of locoregional recurrence will be tracked as the primary endpoint of the study. The LUMINA trial, being run by investigators in Canada, is a prospective cohort study evaluating the risk of local recurrence after breast conserving surgery and adjuvant endocrine therapy for women with Luminal A subtyped breast cancer. With a target accrual of 500 women, patients with Luminal A subtype breast cancer (as defined by ER, PR, HER2, and Ki-67 staining, not PAM50 assessment) will be treated with endocrine therapy (tamoxifen or aromatase inhibitor) for five years and will not be treated with breast irradiation. The primary endpoint is local recurrence at 5 years and patients will be followed up for 10 years with other endpoints, including new primary cancers and event-free and overall survival, also being measured. Finally, investigators in the UK are looking at whether IHC4 testing (which combines protein expression of estrogen receptor, progesterone receptor, HER2, and Ki67) in addition to clinical factors can be used to identify patients at low risk for local recurrence in the absence of breast radiotherapy such that radiation can be safely omitted. This trial, called the PRIMETIME trial, is a prospective biomarker-directed case-cohort study in which the IHC4 + C score determines whether radiotherapy is recommended. In this trial, 2,400 patients will be recruited to yield 1550 patients to be treated without radiotherapy. As with the IDEA trial, 5-year local control is the primary endpoint with plans to follow patient outcomes for 10 years [44]. In addition to these trials, various groups are working to develop radiation omission signatures specific to breast cancer patients, and in the coming 5–10 years the results of these and other planned studies will determine whether molecularly based “risk stratification” signatures are able to identify patients for whom adjuvant radiation treatment is unnecessary.

## 5. “Pan-Cancer” Signatures

Rather than developing a breast cancer-specific signature of radiation response, some groups have focused on the development of a “pan-cancer” genomic signature of radiation response. One of the first reports was from a group at the Moffitt Cancer Center. In these initial studies, cell line sensitivity to ionizing radiation was evaluated across 35 and then 48 cell lines in the NCI-60 panel of cancer cell lines [12, 45]. Genes associated with intrinsic radiation response were then identified and formed the basis of the subsequently developed radiation sensitivity index (RSI), which has been refined to a 10-gene, RNA expression based signature. After additional modifications of the signature, the group has assessed the utility of RSI in various disease types with varying levels of utility identified [8, 29, 30, 46]. A similar approach was utilized by investigators at Columbia University that utilized the same NCI-60 cell line panel to identify radiation response-associated genes [47]. Like the Moffitt group, they identified genes whose basal expression was different between the radiosensitive and radioresistant cell lines and identified a total of 36 genes differentially expressed between these two groups. Unlike the Moffitt group, however, they further identified genes whose expression changed after radiation treatment and were associated with survival after varying doses of radiation treatment. Interestingly, they found that the genes induced (by RNA expression profiling) by radiation were remarkably consistent between tumor types and were a function of p53 status, suggesting an underlying conserved set of genes responsible for responding to genotoxic stress. Comparing the gene lists identified by the Moffitt and Columbia groups, there was surprisingly no overlap between the genes identified in these studies, suggesting that the response to radiation treatment (at least in terms of RNA expression) may be complicated and differs under basal and genotoxic conditions.

Additional genomic classifiers and signatures predictive of response to radiation are currently being developed for prostate, lung, rectal, anal, glioblastoma, and head and neck cancers. As with signatures for breast cancer, these classifiers will need to be externally validated using phase III trials prior to adoption into clinical practice, and it remains to be seen whether a more global “pan-radiation” response signature (discussed previously) can be developed and validated for use in women with breast cancer.

## 6. Tailored Radiation Signatures

In an effort to move towards truly personalized radiation treatment, efforts are also underway to utilize the genomic and transcriptomic information from a woman's own tumor to determine, on an individual patient level, whether radiation is likely to be effective and, if so, what dose may be sufficient for this patient. A recent provocative publication suggests that there may be methods available to achieve this level of personalization. As was discussed earlier, the Moffitt group developed a radiation sensitivity index (RSI) to determine the intrinsic sensitivity of tumors to ionizing radiation [12]. Expanding upon that initial work and to determine ways in which to tailor radiation doses across differing cancer histologies (including invasive ductal carcinoma), Scott et al. recently reported results of their effort to develop a genomic-adjusted radiation dose (GARD) framework as the basis for future radiation trial design [9]. To develop GARD, the investigators incorporated their previously developed radiation sensitivity index (RSI) with the linear-quadratic model to derive the genomic-adjusted radiation dose and used this to calculate the GARD-value for over 8000 tumor samples. In this retrospective analysis, GARD appeared to predict clinical outcome in several cancer types. While limited by an incomplete evaluation in patients who have not received radiation treatment (to evaluate whether GARD has more than just prognostic value) and lack of external validation, this approach does hold promise and is worthy of continued investigation. It also underscores the potential utility of a genomic-based radiation response signature that may be used to assess, prior to the initiation of treatment, the likelihood of tumor response and control of disease with radiation treatment.

In addition to the development of GARD, a more comprehensive assessment was done by investigators at the Cleveland Clinic and the Broad Institute who sought to understand the genetic basis of DNA damage response after radiotherapy [14]. In this study, investigators profiled the radiation response of over 500 cell lines to ionizing radiation and showed that sensitivity to radiation is characterized by significant genetic variation across and within lineages (in contrast to the Moffitt group, whose work suggests that a 10-gene signature may be sufficient to describe radiation response across all disease types and tumor lineages). In addition to identifying genes whose expression was associated with response, they also identify somatic copy number alterations and gene mutations that correlate with the radiation survival. This work offers arguably a more comprehensive (and a more complicated) picture of the genomic basis of radiation response and may serve as a foundation for future signature development to predict response of various tumors to radiation treatment across all cancers.

## 7. Barriers to Clinical Adoption

Although numerous in development, molecular signatures predictive of radiation response have been slow to gain clinical traction. Reasons for their slow uptake include a lack of external validation, feasibility concerns in a clinical setting, and nonspecificity for the clinical situation at hand. As was noted previously, most of the radiation response signatures generated to date have failed external validation in cohorts in which they were not trained. This may reflect factors such as variation in the clinic-pathologic characteristics of the patients represented in these cohorts, differences in radiation treatment techniques and doses, and imbalances between the treated and untreated patients. It may also reflect the heterogeneous biological underpinnings of radiation response in breast cancer, suggesting that these signatures are unable to account for the complexity of the radiation response. Additionally, variation in gene expression measurements and normalization between fresh frozen, formalin fixed, and paraffin embedded tissues has proved challenging as previously developed tests have transitioned from research endeavor to clinical implantation. This same challenge has limited the scalability of these molecular signatures. Furthermore, translating research-lab derived molecular signatures into a clinically available test is not a trivial task. Aside from barriers with cross-platforming of molecular tests, developing standard operating procedures and certification in accordance with Clinical Laboratory Improvement Amendments (CLIA) is often costly and fraught with regulation meant to safeguard patients. Taken collectively, these reasons help explain why so many tests have been reported in the academic literature but so few have successfully been translated into clinical practice.

## 8. Future Directions

Much as the development of molecularly based signatures (OncotypeDx, MammaPrint, Prosigna, etc.) has revolutionized the decision-making process surrounding the need for adjuvant chemotherapy in women with early-stage breast cancer, the development of prognostic and predictive signatures to determine the need and efficacy of radiation for women with breast cancer holds similar promise. While preliminary efforts to develop these signatures has been encouraging, much work remains in order to successfully translate these signatures into the clinic. Whether RSI, OncotypeDX for Breast DCIS, GARD, RadiotypeDx, or other as-yet developed signatures will gain similar traction remains to be seen. Ultimately, for any of these tests to be translated into the clinic it will require demonstration of their accuracy and reproducibility as a test and perhaps more importantly demonstration within the context of clinical trials of the utility of these tests at improving outcomes for women with breast cancer. While not yet realized, the ongoing development of these signatures holds much promise as the field seeks to finally realize “personalized medicine” as it relates to radiation treatment for women with breast cancer.
